# Valorisation of Culled Iberian Sows Through the Montanera System: Immunocastration, Welfare and Sustainable Production

**DOI:** 10.3390/ani16010152

**Published:** 2026-01-05

**Authors:** Javier García-Gudiño, Carmen Barraso, Francisco I. Hernández-García, Montaña López-Parra, Mercedes Izquierdo, Isabel Blanco-Penedo

**Affiliations:** 1 Animal Production, Centre of Scientific and Technological Research of Extremadura, 06187 Guadajira, Spain; javier.garciag@juntaex.es (J.G.-G.);; 2 Meat Quality, Centre of Scientific and Technological Research of Extremadura, 06187 Guadajira, Spain; 3 Department of Animal Sciences, University of Lleida, 25918 Lleida, Spain; isabel.blancopenedo@udl.cat; 4 Department of Clinical Sciences, Swedish University of Agricultural Sciences, 750 07 Uppsala, Sweden

**Keywords:** *dehesa* ecosystem, natural resources, castration alternatives, animal behaviour, meat quality

## Abstract

Despite their substantial contributions to herd productivity, culled sows typically receive minimal economic valorisation. This study evaluated the feasibility of finishing culled Iberian sows under free-range conditions (*montanera* finishing system) and assessed the effects of immunocastration on productivity, welfare, and meat quality. Thirty-six culled Iberian sows were assigned to two treatments: entire sows (*n* = 18) and immunocastrated sows (*n* = 18). Following a maintenance phase and a pre-finishing transition, animals were finished under *montanera* conditions with ad libitum access to acorns and pasture. Productive traits, welfare indicators, carcass traits, and meat quality parameters were evaluated. Immunocastration effectively suppressed reproductive function without compromising productive traits or meat quality in culled Iberian sows. Both groups exhibited comparable body weight evolution, carcass characteristics, and meat quality attributes consistent with Iberian standards. The results indicate a clear potential for value generation, transforming animals from minimal-value culling categories to premium *montanera* products. This integrated approach combining immunocastration with traditional extensive finishing systems provides a viable way to add value to culled sow valorisation within circular economy frameworks, addressing economic, environmental, and welfare challenges of sustainable livestock production.

## Simple Summary

Breeding sows are typically sold at very low prices when culled. This study investigated whether these animals could generate higher value when finished in the traditional acorn-based system for premium Iberian pork. We also investigated immunocastration, which temporarily stops reproductive activity and could improve management and welfare. Thirty-six Iberian sows were divided into two groups: one vaccinated and one non-vaccinated. Following an adaptation period, all animals grazed on acorns and natural pasture before slaughter. Results showed the vaccination did not affect growth or meat quality, and both groups produced excellent meat suitable for premium products. Economic analysis revealed that this approach can transform low-value animals into high-value products, providing farmers with a profitable strategy that also benefits animal welfare, reduces environmental impact, and preserves the traditional management of Iberian pig production.

## 1. Introduction

The nexus between animal production and sustainability recognises the economic, social, and cultural value of livestock systems [1,2]. Within this framework, livestock are increasingly emphasised as integral components of agroecosystems [3], providing benefits beyond direct products such as meat, milk, and eggs, notably through ecosystem services. This perspective places livestock at the core of sustainable food systems, and underscores the need to valorise animals throughout their productive lifespan [4]. In pig production, breeding sow performance contributes substantially to herd profitability, but they typically receive limited economic recognition at the end of their reproductive lives.

Despite their essential contribution to herd productivity and long-term system performance, breeding sows are frequently undervalued upon reaching the end of their reproductive lifespan [5], typically at 2–3 years of age [6]. Culled sows generally command lower market prices due to perceived inferior meat quality compared to younger animals [7], leading to their channelling in low-value processed products or export markets. This creates a value gap in which animals that have contributed continuously over extended periods receive minimal economic recognition upon culling. In Iberian pig production, however, the technological processing characteristic of dry-cured products may mitigate age-related quality concerns [8]. This highlights that valorising culled Iberian sows is feasible.

In order to address the aforementioned challenge, it is first necessary to recognise the value of animals across the entire course of their productive lifespan. Pig production must be reframed as a biologically responsible system, in which every stage of production has ethical and economic importance, including the final reproductive phase [9]. Within this framework, immunocastration emerges as a welfare-oriented management tool that facilitates the handling of pre-culled sows during extended fattening periods, whilst eliminating reproductive function without surgery [10]. Beyond its animal welfare benefits, the integration of culled sow management with natural resource utilisation within the dehesa ecosystem presents additional sustainability opportunities [11]. Traditional extensive systems characteristic of Iberian pig production, which rely on acorns and pasture provided ad libitum and a variable stocking density ranging from 0.25 to 1.25 pigs per hectare according to oak tree availability, provide a unique context wherein welfare-compatible reproductive management may complement resource-efficient finishing strategies [12]. The feasibility of these approaches, however, also depends on societal acceptance and consumer perceptions.

As consumer expectations increasingly emphasise longevity, respect for animals, and sustainable resource use [13,14], the present study focuses on the technical and management aspects of valorising culled Iberian sows. Recognising the contributions of breeding animals to agroecosystem resilience—through their genetic value, longevity, and role in maintaining stable production systems—and improving their end-of-life management are both aligned with broader sustainability principles relevant to contemporary livestock systems.

Enhancing the management of culled Iberian sows emerges as a key strategy for improving farm sustainability and animal resource efficiency. However, empirical evidence on this integrated approach remains limited. Therefore, this study aimed to evaluate montanera finishing of culled Iberian sows, assessing the effects of immunocastration on animal welfare and meat quality, as well as the economic and environmental implications of revalorisation within traditional Iberian pig production systems.

## 2. Materials and Methods

### 2.1. Animals and Experimental Design

**Figure 1:**
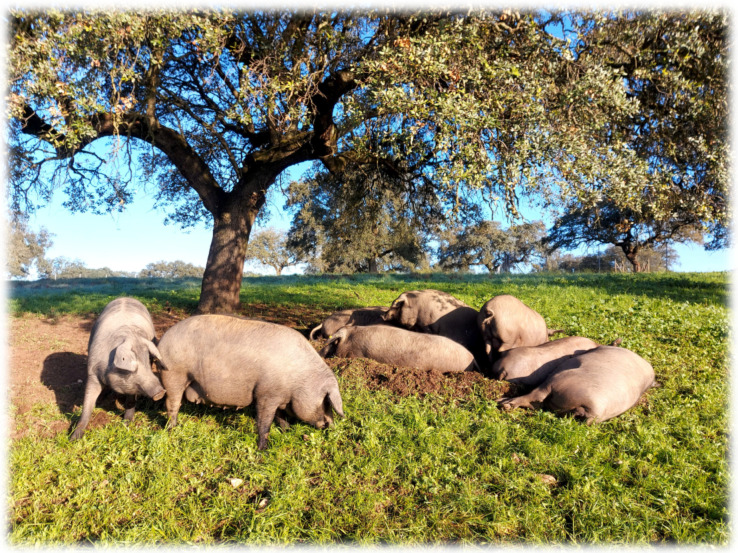
Iberian sows during the finishing period (*montanera*) at the experimental farm Valdesequera (CICYTEX, Extremadura, Spain) in the *dehesa* ecosystem (Photo: Javier García-Gudiño).

Animals were raised at the experimental farm Valdesequera belonging to CICYTEX (Extremadura, Spain; 39°3′13.2″ N, 6°50′45.2″ W; Figure 1). Sows were selected based on routine farm records of low productivity or reproductive failure, as registered by farm veterinary staff (see Table 1). Sows selected for causes other than reproductive disorders were weaned approximately two months before the start of the experiment. All animals were confirmed non-pregnant via transabdominal ultrasonographyprior to the start of the trial.

**Figure 2:**
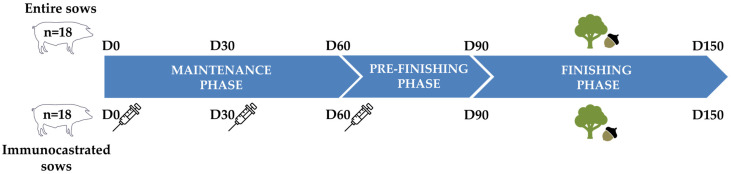
Experimental timeline showing the maintenance phase (D0–D60), pre-finishing phase (D60–D90), and finishing phase (D90–D150) under *montanera* conditions, ending with slaughter at D150. Two groups of culled Iberian sows were compared: entire sows and immunocastrated sows. Immunocastrated sows received three vaccine doses at D0, D30, and D60. The *montanera* finishing phase aligned with natural resource availability (November–January).

As illustrated in Figure 2, the experimental timeline lasted for 150 days, divided into three different phases with specific feeding strategies and management practices. A total of 36 culled Iberian sows were randomly allocated into two experimental groups: entire sows (*n* = 18) and immunocastrated sows (*n* = 18). Animals were randomly assigned to treatments by age, body weight, and culling cause to ensure balanced distribution between groups. The mean age was 2.43 ± 1.19 years in entire sows and 2.53 ± 1.14 years in immunocastrated sows. Initial body weight at day 0 was 140.7 ± 3.7 kg for entire sows and 142.3 ± 3.7 kg for immunocastrated sows, confirming similar baseline characteristics between groups. Both experimental groups were maintained separately throughout the entire experimental period, with continuous access to fresh water and appropriate shelter facilities.

The experimental timeline was structured as follows: (1) maintenance phase (60 days), where all sows received a maintenance diet (Table 2) at 1.4 kg/day/sow, aimed at preserving body weight (BW) whilst minimising feed costs. During this phase, sows in the immunocastration group were administered three doses of a commercial vaccine (Vacsincel^®^, Zoetis, Louvain-la-Neuve, Belgium) at 30-day intervals (day 0, day 30, and day 60), with each dose consisting of the standardised volume of 2 mL per sow. This immunocastration protocol was applied in accordance with the manufacturer’s recommendations and had previously been validated in female Iberian pigs [15]. The experimental timeline continued with (2) pre-finishing phase (30 days), where both experimental groups received 3.0 kg/day/sow of a pre-fattening diet (Table 2) under semi-extensive conditions, providing a progressive transition from the maintenance diet to the subsequent ad libitum feeding period; and (3) finishing phase (60 days), consisting of a fattening period under the *montanera* system (extensive free-range), exclusively consuming natural resources of the *dehesa* ecosystem, including acorns (*Quercus* spp.) and pastures, in accordance with the Iberian Quality Standard [16]. At the end of the trial, animals were transported to a commercial slaughterhouse (96 km distance) using a CICYTEX livestock truck, in accordance with EU regulations. To maintain group separation and ensure identical pre-slaughter conditions, the two groups were transported separately in two consecutive trips on the same morning (approximately 1.5 h each), under comparable environmental conditions (winter season, stable weather).

### 2.2. Growth Measurements, Carcass Traits and Sample Collection

Body weight (BW) was individually recorded at 30-days interval until the start of the finishing phase in both experimental groups (immunocastrated and entire sows). An additional weighing was conducted at the end of the finishing phase to minimise interference with animal management during their time in the *dehesa* ecosystem.

Ultrasound measurements were obtained at the beginning and end of the maintenance phase, and at the beginning and end of the finishing phase. Animals were gently restrained in a crate to minimise movement whilst maintaining a standing position. Scans were performed using an EXAGO ultrasound scanner (IMV Technologies, L’Aigle, France) equipped with a 13 cm, 3.5 MHz linear probe, following validated protocols previously described by Ayuso et al. [17]. Longitudinal images were taken approximately at 7 cm from the dorsal midline at the level of the 10th to the 14th ribs, with probe position adjusted to visualise maximum *Longissimus dorsi* muscle thickness, to assess loin depth and backfat thickness. Transverse scans were performed between the 10th and 11th ribs using a silicone adapter for thoracic curvature to measure loin area. Three images were captured per animal at each measurement site and time point, and measurements were averaged. Image analysis was performed using the BioSoft Toolbox^®^ II for Swine software (Version X, Biotronics Inc., Ames, IA, USA) [18]. All ultrasound measurements were performed by the same trained operator throughout the study to ensure consistency, with the aim of assessing body composition during the fattening phase.

Blood samples were collected via orbital sinus puncture at two time points: at the beginning of the maintenance phase, and at the end of the finishing phase prior to slaughter. Samples were obtained in vacuum tubes, allowed to clot, and centrifuged to obtain serum. Progesterone and estradiol concentrations were determined using a commercial ELISA kit (Progesterone ELISA Kit, ADI-901-011 and 17β-Estradiol ELISA Kit, ADI-900-008; Enzo Life Sciences, Farmingdale, NY, USA) to confirm immunocastration efficacy.

After the slaughter, the weights of carcasses and prime cuts (hams, shoulders, and loins) were recorded. Specifically, carcass weight (CW) was measured post-slaughter and post-dressing. Each hind leg, front leg, and loin was weighed individually after removal. Ham weight (HW), shoulder weight (SW), and loin weight (LW) represent the combined weight of both sides per animal. The prime cut weight (PCW) was calculated as the sum of ham, shoulder and loin weights (PCW = HW + SW + LW). The yields of carcass (CY), hams (HY), shoulders (SY), loins (LY), and total prime cuts (PCY) were calculated using the following equations:

CY = (CW/BW) × 100

HY = (HW/CW) × 100

SY = (SW/CW) × 100

LY = (LW/CW) × 100

PCY = (PCW/CW) × 100

These calculations were performed in accordance with the methodology outlined by García-Gudiño et al. [19] to ensure a standardised approach for evaluating carcass and cut yields. For meat quality analyses, the left loin of each carcass was sampled. Additionally, reproductive organs (uterus and ovaries) were excised and weighed separately from the carcass.

### 2.3. Animal Welfare Assessment

Animal welfare was assessed using the Welfare Quality^®^ protocol for pigs [20], which is structured around four welfare principles: good feeding, good housing, good health, and appropriate behaviour. These principles encompass 12 welfare criteria [21] with approximately 30 individual measures. The complete protocol was evaluated on-farm by a single trained observer who was not blinded to treatment groups. However, the use of standardised scoring criteria and predominantly quantitative measures minimised potential observer bias.

Welfare assessments were conducted at four time points corresponding to key experimental phases: at the beginning and end of the maintenance phase (days 0 and 60), and at the beginning and end of the finishing phase (days 90 and 150). Both experimental groups were housed separately yet under identical management conditions throughout the study period. After data collection, scores for criteria, principles, and the overall classification were computed using the WAFA software (version 3.0) developed by the Welfare Quality^®^ Consortium (INRAE, Paris, France) [22].

### 2.4. Meat Quality Analyses

For each loin sample, instrumental colour and pH values were measured. Colour parameters, including lightness (L*), redness (a*), and yellowness (b*) in the CIE Lab colour space, were determined using a hand-held colorimeter (Minolta CR-300, Konica Minolta, Osaka, Japan), according to the method described by Cassens et al. [23]. pH was determined with a portable puncture pH meter (model HI98163, Hanna Instruments, Woonsocket, RI, USA).

Intramuscular fat (IMF) content was determined by the method of Folch et al. [24], based on extraction with chloroform–methanol (2:1). The solvent was then evaporated, and the extracted fat was weighed. The fatty acid profile of the total lipid extract was determined using the method described by Morrison and Smith [25]. Analysis of fatty acid methyl esters (FAMEs) was conducted using an Agilent 6890 gas chromatograph (Agilent Technologies, Santa Clara, CA, USA) equipped with a flame ionization detector (FID) and a silica column DB-23 (60 m length, 0.25 mm inner diameter, and 0.25 μm film thickness). The injector and detector were held at 250 °C. The column oven temperature was held at 220 °C. The carrier gas was nitrogen at a flow rate of 10 mL/min. FAMEs were quantified using tridecanoic acid as an internal standard. Identification of individual FAMEs was performed by comparing retention times with those of standard FAME mixtures (Supelco 37 Component FAME Mix, Sigma Aldrich, St. Louis, MO, USA). Results were expressed as g/100 g of total FAMEs.

Lipid oxidation was evaluated by the thiobarbituric acid reactive substances (TBARS) assay according to Salih et al. [26], with results expressed as μg malondialdehyde (MDA)/g of sample. Protein oxidation was measured by quantifying carbonyl groups after derivatisation with 2,4-dinitrophenylhydrazine (DNPH) as described by Oliver et al. [27], and expressed as nmol carbonyls/mg protein. Protein content was determined by the Kjeldahl method [28], using a nitrogen-to-protein conversion factor of 6.25.

For texture analysis, loin samples were vacuum-packed, cooked in a water bath at 75 °C for 45 min, and evaluated with a TA-XT2i Texture Analyser (Stable Micro Systems Ltd., Surrey, UK) equipped with a Warner–Bratzler blade, following Combes et al. [29]. Maximum shear force (Fmax) was measured on slices cut perpendicular to muscle fibres. Determinations were repeated 8 times per sample and the data were averaged.

### 2.5. Statistical Analyses

For growth parameters, reproductive hormones, carcass traits, and meat quality variables, data were analysed using one-way analysis of variance (ANOVA) with the GLM procedure in SAS software version 9.4 (SAS Institute Inc., Cary, NC, USA). The statistical model included treatment group as a fixed effect, with individual sows as experimental units. For repeated measurements (body weight, ultrasound parameters, and hormone concentrations), each time point was analysed separately. Prior to analysis, normality and homogeneity of variance were assessed using Shapiro-Wilk and Levene’s tests, respectively. Results are presented as least squares means ± RMSE, with significance set at *p* < 0.05.

For animal welfare data collected via the Welfare Quality^®^ protocol, numerical scores for criteria and principles were presented descriptively without statistical comparison, as assessments were conducted at the group level (one assessment per treatment group per time point), thus precluding statistical analysis.

## 3. Results

### 3.1. Productive Traits and Physiological Response

Body weight evolution during the experimental phases is shown in Table 3. No significant differences were observed between entire and immunocastrated sows across the different BW measurements recorded (*p* > 0.05). Both groups exhibited a progressive increase in BW, particularly during the finishing phase (BW90 and BW150), attributed to the ad libitum feeding on natural resources during this productive phase. This pattern suggests that immunocastration did not adversely affect productive traits related to BW under these experimental conditions.

In vivo carcass traits predicted by ultrasound are presented in Table 4. Loin depth, loin area, and backfat thickness showed no differences between treatment groups across the different ultrasound measurements taken during the study (*p* > 0.05). These results indicate that immunocastration did not alter body composition during the experimental period.

Serum progesterone and estradiol concentrations are presented in Table 5. No significant differences were observed between groups at day 0 (*p* > 0.05). At the end of the *montanera* fattening period (day 150), both progesterone and estradiol concentrations were significantly lower in immunocastrated sows compared to entire sows (*p* < 0.001), confirming effective implementation of the immunocastration protocol.

### 3.2. Welfare Assessment

**Figure 3:**
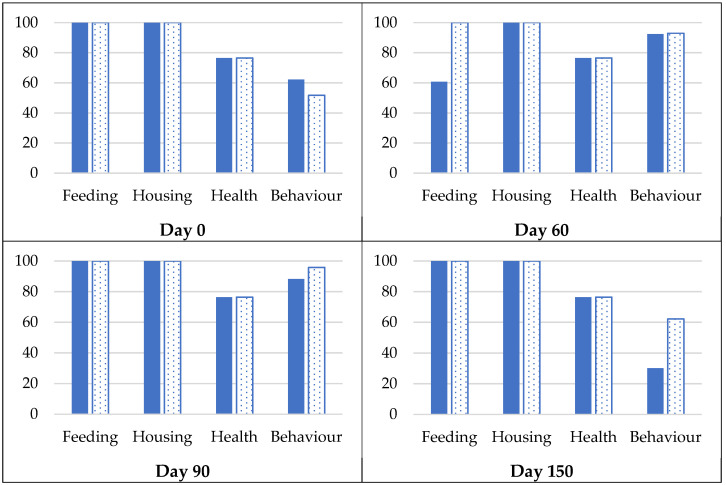
Welfare Quality^®^ principle scores during experimental phases in culled Iberian sows during the experimental phases on days 0, day 60, day 90, and day 150. Scores represent group-level assessments at the start and end of the maintenance phase and at the start and end of the finishing phase, respectively. Feeding: good feeding; Housing: good housing; Health: good health; Behaviour: appropriate behaviour. Blue solid bars: entire sows; blue dotted bars: immunocastrated sows.

Welfare Quality® assessments revealed high welfare standards in both treatment groups throughout the study. Figure 3 shows scores of the four welfare principles: good feeding, good housing, good health, and appropriate behaviour. Whilst scores were generally similar between groups, immunocastrated sows showed notably higher scores for good feeding at the end of the maintenance phase (day 60) and for appropriate behaviour at the end of the finishing phase (day 150). Detailed principle and criteria scores are presented in the Supplementary Materials (Table S1).

### 3.3. Carcass Traits and Meat Quality Attributes

Table 6 presents carcass traits. Immunocastrated sows showed significantly higher carcass yield compared to entire sows (*p* = 0.034). In contrast, shoulder yield was significantly reduced in immunocastrated animals (*p* = 0.031). No statistically significant differences were detected between entire and immunocastrated sows for the recorded weights (carcass, ham, shoulder, and loin) and the remaining calculated yields (prime cut, ham, and loin yields). Reproductive organ weight was 91.1 ± 8.2 g in immunocastrated sows and 656.5 ± 27.6 g in entire sows (*p* < 0.001).

Table 7 shows meat quality attributes. Immunocastrated and entire sows showed no differences in colour, pH, composition (IMF and protein), fatty acid profile, oxidative stability, and tenderness (*p* > 0.05).

## 4. Discussion

A distinctive feature of this work is its focus on culled sows under commercial conditions, where these animals represent a heterogeneous population with variable reproductive histories. Rather than imposing artificial selection criteria, animals were randomly allocated to treatment groups to ensure that baseline variability was evenly distributed. Combined progesterone and estradiol suppression at the end of the study (Table 5), together with the 86% reduction in reproductive tract weight, confirmed effective ovarian suppression in immunocastrated sows. These results are consistent with previous findings in Iberian female pigs demonstrating successful ovarian suppression following GnRH immunization [15], and demonstrate the robustness and practical applicability of this management strategy across the diverse reproductive states naturally present in commercial herds.

### 4.1. Production Performance and Meat Quality

A key consideration in valorising culled sows concerns their production performance and meat quality attributes. This study evaluated these aspects in culled Iberian sows finished under montanera conditions, comparing entire and immunocastrated females

Immunocastration did not significantly affect BW evolution, as evidenced by the absence of significant differences in BW throughout the experimental period (Table 3). Similarly, ultrasound measurements revealed no differences in loin depth, loin area, or backfat thickness between treatment groups at any evaluation time point (Table 4), indicating that muscle development and subcutaneous fat deposition were not altered by immunocastration. These findings are consistent with Macipe et al. [30], who reported no differences in growth performance between entire and immunocastrated Iberian gilts finished under *montanera* conditions, and are aligned with studies showing that immunocastration does not impair growth in female pigs [31,32]. The absence of effects on body composition confirms that, in adult Iberian sows that had already reached their full growth potential, the immunocastration protocol does not interfere with normal productive performance.

Regarding productive parameters measured at slaughter, some differences were observed, though of limited practical significance. The significantly higher carcass yield in immunocastrated sows (76.88% vs. 75.56%, *p* = 0.034) can be partially attributed to the atrophy of the reproductive tract induced by immunocastration [33], as evidenced by the 86% reduction in reproductive organ weight observed in the present study, along with potential differences in gut content. This finding aligns with Gómez-Fernández et al. [34], who reported similar improvements in carcass yield in immunocastrated female Iberian pigs. However, both carcass weight and yields should be interpreted in the context of the larger body size and advanced age of culled sows, which naturally results in lower carcass yield percentages than those observed in younger finishing pigs [19]. The modest reduction in shoulder yield in immunocastrated sows (12.38% vs. 12.80%, *p* = 0.031) likely reflects proportional redistribution rather than absolute reduction, as absolute shoulder weights were similar between groups (*p* = 0.182). This proportional redistribution effect has also been observed by Gómez-Fernández et al. [34] in immunocastrated female Iberian pigs.

Overall, meat quality parameters in both entire and immunocastrated culled sows were consistent with the ranges reported for Iberian pigs finished under *montanera* conditions [35,36,37]. These findings align with Macipe et al. [30], who observed comparable meat quality in entire and immunocastrated Iberian gilts, and with Abreu et al. [38], who demonstrated that extensive rearing systems preserve muscle characteristics in Iberian sows regardless of reproductive or hormonal status, supporting the stability of meat quality across age categories when immunocastration is applied in this production system. Importantly, the quality attributes observed in culled sows of both groups are within the ranges reported in the scientific literature for gilts destined for dry-cured products, demonstrating that both entire and immunocastrated females can produce meat suitable for the high-value Iberian pork market.

Physical and chemical meat quality parameters fell within expected ranges for Iberian pork. Ultimate pH was within the optimal range [39]. Colour coordinates (L*, a*, b*) in both groups were lower than those reported for younger Iberian pigs [35,37], consistent with the advanced age and elevated myoglobin content of culled sows. The reduced yellowness values also align with lower intramuscular fat deposition in these animals. This darker meat colour, characteristic of older Iberian pigs, is valued by consumers as an indicator of authenticity and traditional production [40]. Importantly, immunocastration did not affect any colour coordinate, confirming no impact on meat appearance.

Intramuscular fat content aligned with values reported for *Longissimus dorsi* in similar systems [35,41,42]. Although somewhat lower than the IMF levels typically reported for Iberian pigs finished under extensive free-range (*montanera*) systems, these levels remain within the acceptable range and are sufficient to support the technological qualities of Iberian pork. The lower IMF in culled sows may reflect reduced lipogenesis associated with age and physiological status [43]. Beyond lipid quantity, the fatty acid profile quality is equally critical for Iberian products. The fatty acid composition exhibited a profile characteristic of acorn-finished Iberian pork, with an oleic acid content of 51.03%, which is consistent with the typical *montanera* range [35,36]. The high MUFA content contributes to the distinctive nutritional and sensory properties of Iberian dry-cured products [44]. The similar SFA, MUFA, and PUFA percentages observed in both groups are consistent with previous reports for Iberian pigs [36], confirming that immunocastration did not affect the fatty acid profile resulting from *montanera* feeding.

Both groups exhibited excellent oxidative stability, with very low TBARS values well below rancidity thresholds. This stability likely reflects the beneficial effects of high MUFA content and natural antioxidants from acorns and pasture [35]. Warner–Bratzler shear force values were within the tender range for Iberian pigs [45], indicating excellent tenderness regardless of treatment.

Overall, both entire and immunocastrated culled sows produced meat meeting the physicochemical and sensory standards reported in the literature for high-quality Iberian dry-cured products [35,36,37]. Differences between treatments were minimal, confirming that either strategy is compatible with the production of Iberian products.

### 4.2. Immunocastration and Animal Welfare

When immunocastration is implemented in commercial pig production, consideration should be given to its potential effects on behaviour and social interactions, since the temporary suppression of gonadal function may influence these parameters [46].

Welfare Quality^®^ assessments revealed high welfare standards in both treatment groups across all evaluation time points (Figure 3). Overall, culled sows achieved welfare levels comparable to those previously reported for Iberian pigs in extensive systems [47], demonstrating that appropriately managed culled sows can meet the welfare benchmarks established for Iberian traditional pig production. Immunocastrated sows showed slightly higher scores in specific welfare criteria at particular time points.

Two notable differences between groups emerged at specific time points. At the end of the maintenance phase, immunocastrated sows achieved higher scores in the good feeding principle, particularly in the absence of the prolonged hunger criterion (Table S1). This improvement likely reflects reduced agonistic interactions around feeding [48,49,50], as immunocastrated sows, which no longer experience oestrous cycles [51], displayed calmer behaviour and less competition for restricted feed rations. Entire sows may show increased activity and restlessness during oestrus, which may impair feed intake. The absence of oestrus-related activity and social tension may have facilitated more equitable access to feed within the group.

At the end of the finishing phase, immunocastrated sows scored higher in appropriate behaviour, specifically in the expression of social behaviours criterion (Table S1). This difference likely reflects the elimination of oestrous-related behaviours [51,52], which are assessed as social disruptions in welfare protocols.

Overall, the comprehensive Welfare Quality^®^ assessments conducted at four time points confirm that immunocastration does not compromise animal welfare in culled Iberian sows. Both treatment groups maintained high welfare scores throughout the study, and immunocastrated sows showed modest behavioural advantages related to the elimination of oestrus-associated activity [52]. From an animal welfare perspective, these findings reinforce the suitability of immunocastration as a welfare-friendly and legally compliant alternative to surgical castration [53].

Behavioural and welfare assessments conducted using the Welfare Quality^®^ protocol were performed at the group level without pen-level replication. Therefore, the behavioural results are intended to provide a descriptive overview of welfare status and should be interpreted with appropriate caution.

### 4.3. Economic and Sustainability Aspects

**Figure 4:**
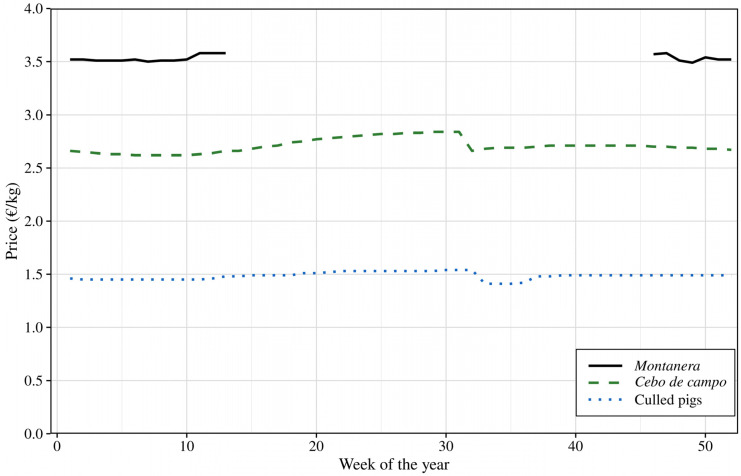
Mean weekly prices (€/kg) for Iberian pig categories over 2022–2024. *Montanera*: free-range finishing with pasture and acorns; *Cebo de campo*: outdoor finishing with concentrate, may include pasture; Culled pigs: animals at the end of their reproductive life. Data represent average prices from Iberian pig markets in Spain.

The economic transformation achieved through this valorisation strategy is substantial. Culled sows sold directly for slaughter typically command low market prices [7]. However, animals finished under *montanera* conditions achieve market prices more than double those of animals sold for direct slaughter (Figure 4), thereby converting an underutilised resource into a high-value product. Meat quality remained within acceptable commercial ranges, supporting market viability [35,36,38]. This economic revalorisation is further enhanced by the low direct feeding costs under the *montanera* system, as animals consume natural resources (acorns and pasture) directly from the *dehesa* [11]. Although indirect costs such as land use and extended finishing periods must be considered, the elimination of purchased feed represents a substantial economic advantage. The combination of high market prices, consistent product quality and low finishing costs demonstrates the economic viability of culled sow valorisation within the Iberian production system.

The sustainability advantages of *montanera* fattening extend beyond economic revalorisation. This extensive system utilises natural resources with minimal purchased feed requirements, resulting in lower environmental impacts compared to other Iberian pig production systems [54,55,56]. However, as illustrated by seasonal price patterns (Figure 4), *montanera* is constrained by resource availability, typically limited to November–March, depending on acorn crop yield and climatic conditions [11]. Outside this period, alternative fattening systems such as *cebo de campo* within the same *dehesa* ecosystem, combining pasture grazing with compound feed supplementation [16], constitute the primary fattening alternative when *montanera* resources are unavailable [57]. Further research in these alternative systems is warranted to assess their economic outcomes and product quality, thus supporting potential year-round implementation of culled sow valorisation strategies.

Beyond production system optimization, commercial viability depends on maintaining product quality standards. The use of reproductive females for high-quality Iberian products has precedent in traditional production systems [58], demonstrating that reproductive status does not compromise meat quality. Consumer acceptance of pork products is increasingly influenced by production management practices, particularly those related to animal welfare [59,60]. Immunocastration, as a welfare-oriented management tool, may align with these consumer preferences, thereby supporting the economic feasibility of culled sow valorisation. A potential limitation in the valorisation of meat from culled Iberian sows is the presence of sexual odour, which, although considerably less intense than in intact males, has been occasionally reported in adult sows [60]. However, this aspect primarily affects fresh meat consumption. Notably, culled Iberian sows are predominantly destined for dry-cured products, where the extended curing and maturation processes substantially reduce or eliminate any residual odour compounds, making this a minor concern in the traditional Iberian production chain.

Importantly, immunocastration in culled sows presents a sustainability-oriented approach beyond welfare or meat quality considerations alone [61,62], potentially enhancing consumer acceptance. Future research should prioritise comprehensive sensory analyses and consumer studies to evaluate product perception, acceptance, and willingness-to-pay for meat from valorised culled sows [63,64]. Addressing these aspects will clarify the full commercial potential and social viability of culled sow valorisation within the Iberian pig sector.

## 5. Conclusions

This study demonstrates that finishing culled Iberian sows under *montanera* conditions is a viable and high-value valorisation strategy that addresses economic efficiency, environmental sustainability, and improved welfare. Transitioning animals from low-value culling categories to premium *montanera* quality products reveals that culled sows constitute an underutilised resource within Iberian pig production systems. The use of natural *dehesa* resources (acorns and pasture) enabled the production of high-quality meat with Iberian pork standards whilst minimising environmental impact. Additionally, immunocastration proved effective in suppressing reproductive function without compromising welfare, confirming it as a practical alternative to conventional culling.

This approach redefines resource management in pig production by showing that even the final stage of a breeding animal’s productive cycle can contribute economically rather than representing a loss. The successful integration of modern reproductive management tools with traditional extensive finishing systems shows how livestock production can adapt to contemporary sustainability demands without compromising cultural heritage or product quality. Overall, this study establishes a concrete pathway toward more sustainable and resource-efficient livestock systems through improved valorisation of animals traditionally considered as waste products.

## Figures and Tables

**Table 1:** Distribution of culling causes in Iberian sows used in the study (*n* = 36).

Cause of Culling	Number of Sows	Percentage (%)
Mammary disorders	13	36.1
Reproductive disorders	10	27.8
Suboptimal litter size	9	25.0
Old age	4	11.1

Mammary disorders: udder inflammation or malformation; Reproductive disorders: infertility or repeat breeding; Suboptimal litter size: <6 piglets per litter; Old age: >7 years old.

**Table 2:** Ingredients and nutrient composition of concentrates for culled Iberian sows during the experiment.

	Maintenance Diet	Pre-Finishing Diet
**Ingredients (g/kg)**		
Barley	443	368
Wheat	113	475
Corn	100	108
Wheat bran	215	
Sunflower meal	85.0	
Lard ^1^	4.00	15.0
Calcium carbonate	18.5	15.0
Sodium chloride	5.00	5.00
Dicalcium phosphate	5.50	
Vitamin–Mineral premix ^2^	6.00	9.00
Technological additive ^3^	5.00	5.00
**Nutrient composition (g/kg)**		
Dry matter	889	892
Ash	56.9	36.1
Crude protein	118	91.7
Crude fat	24.8	31.5
Crude fibre	71.3	31.0
Net energy (kcal/kg)	2144	2437

^1^ Product of Intexur S.L. (Higuera la Real, Spain). Fatty acid profile (%): myristic acid (C14:0) 1.4, palmitic acid (C16:0) 25.0, palmitoleic acid (C16:1) 2.8, margaric acid (C17:0) 0.3, margaroleic acid (C17:1) 0.3, stearic acid (C18:0) 12.8, oleic acid (C18:1) 47.2, linoleic acid (C18:2) 7.4, α-linolenic acid (C18:3 n-3) 0.5, arachidic acid (C20:0) 0.2, gadoleic acid (C20:1) 1.1, eicosadienoic acid (C20:2 n-6) 0.4, dihomo-γ-linolenic acid (C20:3 n-6) 0.1, arachidonic acid (C20:4 n-6) 0.1, eicosatrienoic acid (C20:3 n-3) 0.1, docosapentaenoic acid (C22:5 n-3, DPA) 0.1. Other fatty acids < 0.1%. ^2^ Vitamin–mineral premix (Nutrición y Gestión S.L., Lobón, Spain) providing vitamins A, D_3_, E and B-complex; trace elements (Fe, Cu, Mn, Zn, I, Se); enzymes including phytase, β-glucanase and xylanase; probiotics (*Bacillus licheniformis* and *B. subtilis*); and amino acids (lysine, methionine, threonine), including a specialized formulation for Iberian pigs. ^3^ Technological additive to balance the diet and meet nutrient requirements.

**Table 3:** Effect of immunocastration on the body weight (BW, kg) of culled Iberian sows during the experimental phases.

Variable	Entire Sows *n =* 18	Immunocastrated Sows *n =* 18	*p* Value
BW0	140.7 ± 3.91	142.3 ± 3.86	0.759
BW30	142.8 ± 3.81	144.2 ± 3.43	0.786
BW60	141.0 ± 3.54	140.2 ± 3.52	0.878
BW90	162.4 ± 3.29	163.5 ± 3.55	0.834
BW150	203.8 ± 3.25	202.5 ± 3.67	0.793

Body weight (BW) measured at day 0 (BW0), day 30 (BW30), and day 60 (BW60) corresponding to the start, middle, and end of the *maintenance phase* (coinciding with vaccine doses in immunocastrated sows), and at day 90 (BW90) and day 150 (BW150) corresponding to the start and end of the *finishing phase*. Values are expressed as mean ± standard error of the mean (SEM).

**Table 4:** Effect of immunocastration on carcass traits predicted in vivo by ultrasound at the 10th rib level during experimental phases in culled Iberian sows.

Variable	Entire Sows *n =* 18	Immunocastrated Sows *n =* 18	*p* Value
***Day 0***			
Loin depth (cm)	4.41 ± 0.17	4.62 ± 0.16	0.387
Loin area (cm^2^)	26.48 ± 0.88	25.48 ± 0.75	0.396
Back fat (cm)	2.87 ± 0.39	2.66 ± 0.17	0.622
***Day 60***			
Loin depth (cm)	4.58 ± 0.20	4.36 ± 0.12	0.333
Loin area (cm^2^)	25.04 ± 0.98	23.84 ± 0.55	0.278
Back fat (cm)	2.56 ± 0.23	2.70 ± 0.14	0.612
***Day 90***			
Loin depth (cm)	4.61 ± 0.14	5.32 ± 0.16	0.282
Loin area (cm^2^)	25.43 ± 0.58	25.71 ± 0.60	0.742
Back fat (cm)	3.09 ± 0.21	3.23 ± 0.21	0.634
***Day 150***			
Loin depth (cm)	5.66 ± 0.12	5.61 ± 0.13	0.753
Loin area (cm^2^)	29.62 ± 0.76	29.60 ± 0.74	0.990
Back fat (cm)	4.92 ± 0.29	4.58 ± 0.14	0.295

Measurements taken at day 0 (start of *maintenance phase*), day 60 (end of *maintenance phase*), day 90 (start of *finishing phase*), and day 150 (end of *finishing phase*). Values are expressed as mean ± standard error of the mean (SEM).

**Table 5:** Serum progesterone and estradiol concentrations in culled Iberian sows.

Variable	Entire Sows *n =* 18	Immunocastrated Sows *n =* 18	*p* Value
***Day 0***			
Progesterone (pg/mL)	320.5 ± 61.35	244.9 ± 55.8	0.369
Estradiol (pg/mL)	55.30 ± 7.06	50.61 ± 7.36	0.649
***Day 150***			
Progesterone (pg/mL)	241.0 ± 49.48	17.99 ± 1.03	<0.001
Estradiol (pg/mL)	51.07 ± 6.68	22.75 ± 2.84	<0.001

Measurements taken at day 0 and day 150 (end of *finishing phase*). Values are expressed as mean ± standard error of the mean (SEM).

**Table 6:** Effect of immunocastration on carcass traits in culled Iberian sows.

Variable	Entire Sows *n =* 18	Immunocastrated Sows *n =* 18	*p* Value
Carcass weight (kg)	154.1 ± 3.35	155.7 ± 3.53	0.699
Carcass yield (%)	75.56 ± 0.55	76.88 ± 0.43	0.034
Prime cut yield (%)	33.71 ± 0.30	32.80 ± 0.32	0.050
Ham weight (kg)	13.94 ± 0.20	13.84 ± 0.23	0.725
Ham yield (%)	18.13 ± 0.32	17.78 ± 0.21	0.216
Shoulder weight (kg)	9.84 ± 0.11	9.63 ± 0.13	0.182
Shoulder yield (%)	12.80 ± 0.16	12.38 ± 0.18	0.031
Loin weight (kg)	2.14 ± 0.05	2.05 ± 0.04	0.143
Loin yield (%)	2.79 ± 0.07	2.65 ± 0.12	0.240

Values are expressed as mean ± standard error of the mean (SEM).

**Table 7:** Effect of immunocastration on colour parameters, pH, proximate composition, oxidative stability and texture of the *longissimus dorsi* muscle in culled Iberian sows.

Variable	Entire Sows *n =* 18	Immunocastrated Sows *n =* 18	*p* Value
L*	33.64 ± 0.93	33.83 ± 1.21	0.862
a*	10.19 ± 0.49	11.11 ± 0.96	0.255
b*	2.56 ± 0.22	3.01 ± 0.34	0.195
pH	5.53 ± 0.04	5.55 ± 0.06	0.059
IMF (%)	5.57 ± 0.37	5.78 ± 0.66	0.703
Protein (%)	23.17 ± 0.30	22.82 ± 0.38	0.359
SFA (%)	34.99 ± 0.51	34.76 ± 0.57	0.748
MUFA (%)	56.30 ± 0.62	57.08 ± 0.50	0.324
PUFA (%)	8.70 ± 0.30	8.23 ± 0.31	0.232
Lipid oxidation (mg MDA/kg)	0.05 ± 0.00	0.04 ± 0.00	0.059
Protein oxidation (nmol carbonyl/mg protein)	1.70 ± 0.09	1.50 ± 0.08	0.076
Fmax (N)	6.33 ± 0.54	5.28 ± 0.65	0.124

Colour: L* (lightness); a* (redness); b* (yellowness). IMF: intramuscular fat. SFA: saturated fatty acids. MUFA: monounsaturated fatty acids. PUFA: polyunsaturated fatty acids. Fmax: Maximum shear force. Values are expressed as mean ± standard error of the mean (SEM).

## Data Availability

Dataset available from the authors upon request.

## Supplementary Materials

The following supporting information can be downloaded at https://www.mdpi.com/article/10.3390/ani16010152/s1. Table S1: Detailed Welfare Quality^®^ criteria and principle scores during experimental phases in culled Iberian sows.

## Abbreviations

The following abbreviations are used in this manuscript:

BW	Body weight
CW	Carcass weight
HW	Ham weight
SW	Shoulder weight
LW	Loin weight
PCW	Prime cut weight
CY	Carcass yield
HY	Ham yield
SY	Shoulder yield
LY	Loin yield
PCY	Prime cut yield
IMF	Intramuscular fat
FAMEs	Fatty acid methyl esters
