# Supplementary material for: Valorisation of Culled Iberian Sows Through the Montanera System: Immunocastration, Welfare and Sustainable Production
Source: Animals (Basel). 2026 Jan 5;16(1):152. doi: 10.3390/ani16010152 (PMC12785054; doi:10.3390/ani16010152)
Supplement: Supplementary file 1 [file animals-16-00152-s001.zip › animals-4034488-supplementary.pdf]

Table S1. Detailed Welfare Quality® criteria and principle scores during experimental phases in culled Iberian sows.

	Day 0		Day 60		Day 90		Day 150	
	**EN**	**IC**	**EN**	**IC**	**EN**	**IC**	**EN**	**IC**
**Good feeding**								
Absence of prolonged hunger	100	100	45.48	100	100	100	100	100
Absence of prolonged thirst	100	100	100	100	100	100	100	100
**Good housing**								
Comfort around resting	100	100	100	100	100	100	100	100
Thermal comfort	100	100	100	100	100	100	100	100
Ease of movement	100	100	100	100	100	100	100	100
**Good health**								
Absence of injuries	100	100	100	100	100	100	100	100
Absence of diseases	74.13	74.13	74.13	74.13	74.13	74.13	74.13	74.13
Absence of pain induced by management procedures	100	100	100	100	100	100	100	100
**Appropriate behaviour**								
Expression of social behaviours	100	100	100	100	100	100	0	100
Expression of other behaviours	50.24	38.43	88.36	88.21	75.75	92.6	49.39	47.83
Good human-animal relationship	100	100	100	100	100	100	100	100
Positive emotional state	60.3	46.46	94.63	95.95	99.76	98.17	77.51	63.68
**Principles**								
Good feeding	100	100	60.74	100	100	100	100	100
Good housing	100	100	100	100	100	100	100	100
Good health	76.46	76.46	76.46	76.46	76.46	76.46	76.46	76.46
Appropriate behaviour	62.22	51.71	92.53	92.92	88.27	95.81	30.12	62.25
**Global assessment**	Excellent	Excellent	Excellent	Excellent	Excellent	Excellent	Enhanced	Excellent

Assessments conducted at day 0 (start of *controlled-feeding phase*), day 60 (end of *controlled-feeding phase*), day 90 (start of *finishing phase*), and day 150 (end of *finishing phase*). Scores represent group-level evaluations based on Welfare Quality® protocol. Global assessment categories based on protocol thresholds. EN: entire sows; IC: immunocastrated sows.
